# miR-200c dampens cancer cell migration via regulation of protein kinase a subunits

**DOI:** 10.18632/oncotarget.4381

**Published:** 2015-06-29

**Authors:** Florian Christoph Sigloch, Ulrike Christina Burk, Martin Lothar Biniossek, Thomas Brabletz, Oliver Schilling

**Affiliations:** ^1^ Institute of Molecular Medicine and Cell Research, Albert-Ludwigs-University Freiburg, Freiburg, Germany; ^2^ Faculty of Biology, Albert-Ludwigs-University Freiburg, Freiburg, Germany; ^3^ BIOSS Centre for Biological Signaling Studies, University of Freiburg, Freiburg, Germany; ^4^ Experimental Medicine I, Nikolaus-Fiebiger-Center for Molecular Medicine, University Erlangen-Nürnberg, Erlangen, Germany; ^5^ German Cancer Consortium (DKTK) and German Cancer Research Center (DKFZ), Heidelberg, Germany

**Keywords:** miR-200c, miRNA target cluster, PKA, cofilin, proteomics

## Abstract

Expression of miR-200c is a molecular switch to determine cellular fate towards a mesenchymal or epithelial phenotype. miR-200c suppresses the early steps of tumor progression by preventing epithelial-mesenchymal transition (EMT) and intravasation of tumor cells. Unraveling the underlying molecular mechanisms might pinpoint to novel therapeutic options. To better understand these mechanisms it is crucial to identify targets of miR-200c. Here, we employ a combination of quantitative proteomic and bioinformatic strategies to identify novel miR-200c targets. We identify and confirm two subunits of the central cellular kinase protein kinase A (PKA), namely PRKAR1A and PRKACB, to be directly regulated by miR-200c. Notably, siRNA-mediated downregulation of both proteins phenocopies the migratory behavior of breast cancer cells after miR-200c overexpression. Patient data from publicly accessible databases supports a miR-200c-PKA axis. Thus, our study identifies the PKA heteroprotein as an important mediator of miR-200c induced repression of migration in breast cancer cells. By bioinformatics, we define a miRNA target cluster consisting of PRKAR1A, PRKAR2B, PRKACB, and COF2, which is targeted by a group of 14 miRNAs.

## INTRODUCTION

Epithelial-mesenchymal transition (EMT) describes a reversible process which converts adherent, polarized epithelial cells with specialized cell-cell junctions into motile mesenchymal cells [1]. It occurs physiologically during embryogenesis and contributes pathologically to cancer metastasis, the major cause of death in cancer patients [2–4]. Transient rounds of EMT and mesenchymal-epithelial transition (MET) allow tumor cells to acquire increased invasive and migratory abilities for dissemination from the primary tumor and colonization of secondary sites [4–7]. To understand the metastatic dissemination of cancer cells it is crucial to analyze cell migration. Migration is regulated by various signaling pathways and involves complex molecular and cellular processes such as remodeling of cell-cell and cell-matrix adhesion and restructuring of the actin cytoskeleton [8].

Micro RNAs (miRNAs) are small non-coding RNAs, which post-transcriptionally regulate the expression of protein-coding genes [9, 10]. Certain miRNAs have been linked to tumorigenesis (oncomirs) [11–16]. miRNAs of the miR-200 family play an important role in preventing EMT induction by forming reciprocal negative feedback loops with the EMT inducing transcription factors ZEB1 and ZEB2 therefore stabilizing epithelial characteristics and preventing cancer cell migration and invasion [17–19].

Our work focuses on the miR-200 family member miR-200c, which was identified to play a pivotal role in reducing breast cancer cell migration and invasiveness [20, 21]. miR-200c also exerts functions independent of ZEB1/E-cadherin regulation [22], e.g. by direct targeting of actin-binding proteins [21, 23] or the transport protein SEC23A [24].

miRNA mediated silencing of target mRNAs is achieved by two distinct mechanisms: miRNA binding to a 3′UTR binding site leads to target mRNA degradation or translational inhibition [9, 25, 26]. Both mechanisms result in reduced protein abundance, which may or may not be accompanied by reduced mRNA levels [27]. Previous miR-200c target screens mostly rely on transcriptomic data [21, 23, 28], while data on protein level are scarce. However, as standard transcriptomic target screens cannot detect reduced protein abundance following translational repression, complementary proteomic approaches are essential. In order to identify novel miR-200c targets on protein level, we performed a quantitative proteomic profiling of the highly invasive breast cancer cell line MDA-MB-231 after treatment with miR-200c.

We identify and confirm several known and novel targets of miR-200c. We show that miR-200c influences several direct actin-binding proteins, like cofilin-2 (CFL2), fascin (FSCN1), and MARCKS, as well as the cofilin kinase LIM kinase 1 (LIMK1). Importantly, miR-200c also targets regulatory and catalytic subunits of the cAMP-dependent protein kinase A (PKA), which works in an orchestrated manner with LIMK1 to control cofilin phosphorylation, and thus cell migration [29]. We demonstrate that the PKA subunits PRKAR1A and PRKACB are direct targets of miR-200c. Reduction of the subunits leads to impaired migration of breast cancer cells. In the bioinformatic part of our study, we show that PRKAR1A, PRKAR2B, PRKACB, and CFL2 are commonly targeted simultaneously. We describe a group of 14 miRNAs that are able to target all four proteins. We conclude that these proteins form a miRNA target cluster that may play an important role in cell migration.

## RESULTS

### Quantitative proteomics reveals novel targets of miR-200c

miRNAs regulate protein abundance by mRNA degradation or by translational silencing. Transcriptomic analysis is typically not suited to identify targets of translational silencing [30]. In order to identify novel miRNA targets, we used quantitative proteomics to determine changes in protein abundance upon miR-200c transfection in comparison to miR-ctrl transfection. Similar strategies have been successfully employed for miR-376c [31], miR-223 [32], and miR-21 [33], amongst others. Our quantitative proteomics strategy was based on metabolic labeling using SILAC. We chose the highly invasive, mesenchymal-like MDA-MB-231 cell line as an initial model system, since it expresses only low amounts of miR-200c [18]. We have previously demonstrated that transfection of miR-200c into this cell line leads to a mesenchymal-epithelial transition (MET) of the cells, marked by increased E-cadherin expression, reduced migration, and reduced invasion [17]. To analyze the impact of miR-200c on the cellular migratory ability, we performed real-time, as well as end-point measurements of chemotactic transwell migration. Consistent with former experiments [17, 21], migration was strongly impaired upon miR-200c treatment (Supplementary Figure S1A, S1B).

**Figure 1:**
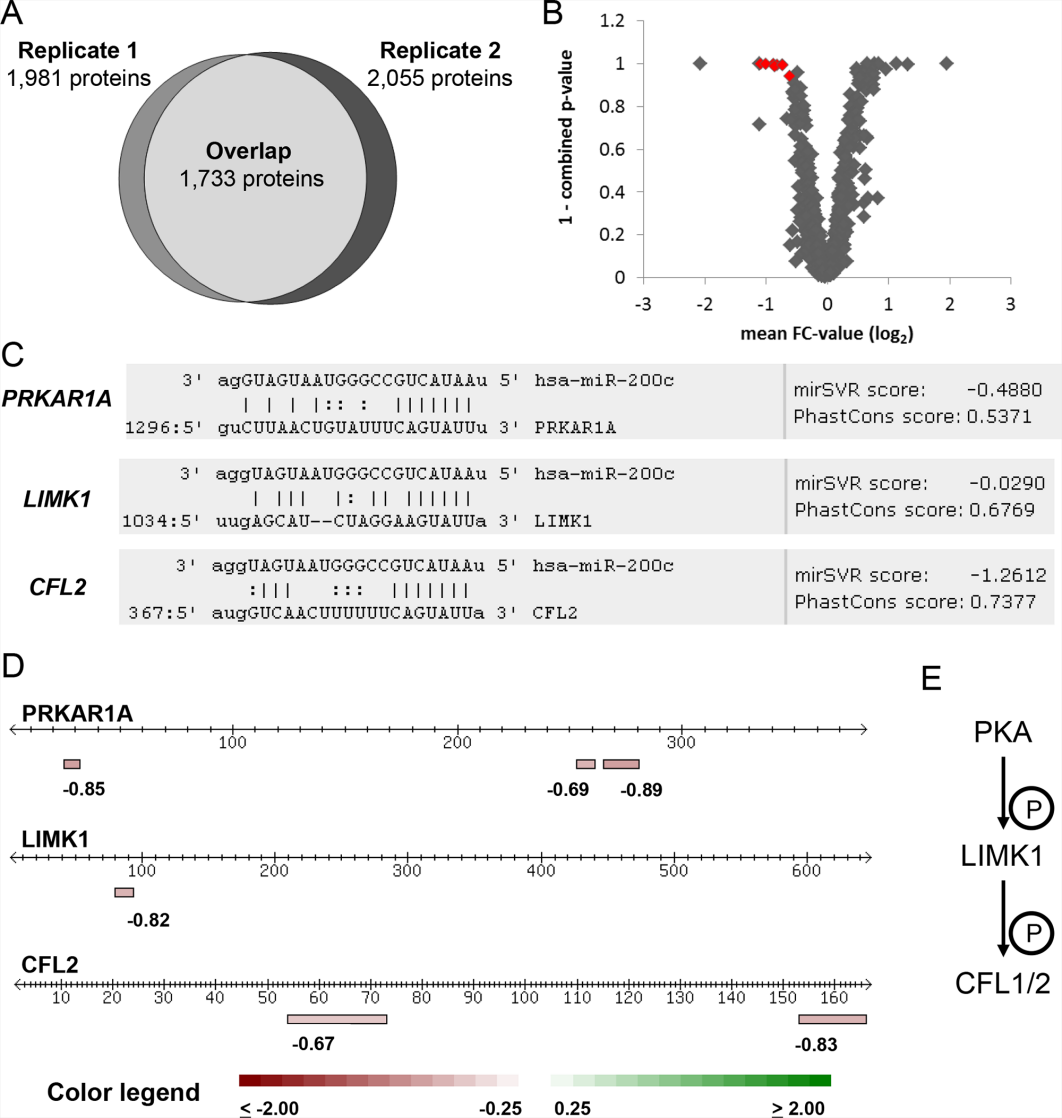
Proteomic profiling identifies miR-200c target candidates **A.** Overlap of protein identification in the two independent biological replicates measured by MS. Each replicate represents a quantitative proteome comparison of MDA-MB-231 cells transfected with miR-200c compared to the non-targeting miR-ctrl. 1,733 proteins were identified and quantified in both replicates. **B.** miR-200c target candidates were identified by a consistent change of protein abundance in both replicates and a combined ASAPRatio *p*-value less than 0.1. A negative FC-value represents a higher abundance in miR-ctrl over miR-200c transfected cells. The plot depicts proteins identified in both replicates. Proteins marked in red fulfilled criteria for further miR-200c target evaluation. **C.** Target candidates were screened for miR-200c binding sites. All target candidates displayed conserved miR-200c binding sites in their mRNA 3′UTR. **D.** Identified peptides used for quantitation of the miR-200c target candidates. Lines represent the protein aminoacid chain, boxes represent the unique peptides used to identify and quantify the proteins. Individual peptide FC-values are given below the boxes, data represent FC-values of one MS experiment. **E.** PKA, LIMK1, and CFL1/2 form a phosphorylation cascade. Cofilins influence actin cytoskeleton remodeling.

For proteomic profiling, two independent biological replicates were measured in distinct mass spectrometry runs, identifying 1,981 and 2,055 proteins, respectively. An incomplete overlap of proteome coverage is an intrinsic characteristic of mass-spectrometry based proteomics [34]. In our approach, 1,733 proteins were identified in both runs (Figure 1A) and considered for further analysis. The observed proteome coverage was within the expected range when using an Orbitrap XL mass spectrometer and SCX prefractionation [35].

We expected potential miR-200c targets to show reduced abundance in both replicates. The software ASAPRatio was used to calculate protein ratios together with a *p*-value to denote the statistical significance of a protein being quantitatively affected [36]. ASAPRatio *p*-values of the two replicates were combined as previously described [35, 37] to yield a merged *p*-value. Proteins were considered as being quantitatively affected if the merged *p*-value was less than 0.1 in agreement with the original ASAPRatio publication [38]. Secondly, a decrease of abundance by more than 25% in both replicates must be observed. Lastly, peptide and protein ratios were manually inspected. Of the 1,733 proteins identified in both replicates, eight proteins fulfilled these criteria (Figure 1B and Table 1).

**Table 1:** miR-200c target candidates identified by proteomic profiling

Gene name	Uniprot	replicate 1		replicate 2		combined *p*-value
		Sequence coverage [%]	Fold change (log_2_) of ASAPRatio	Sequence coverage [%]	Fold change (log_2_) of ASAPRatio	
**CFL2**	Q9Y281	24.7	−1.06	36.7	−0.96	4.57E-04
**FSCN**	Q16658	2.0	−1.03	4.7	−0.45	3.54E-03
**GPX4**	P36969	5.6	−0.47	5.6	−0.77	5.71E-02
**LIMK1**	P53667	2.2	−0.78	2.2	−0.95	1.09E-02
**MARCKS**	P29966	20.8	−0.51	20.8	−1.04	5.13E-03
**PRKAR1A**	P10644	8.9	−0.83	12.3	−0.68	1.39E-03
**SEC23A**	Q15436	5.1	−1.57	6.1	−0.62	6.25E-06
**TBCE**	Q15813	2.3	−1.03	4.6	−0.44	5.55E-03

Out of the eight identified proteins, four proteins were already published to be targeted by miR-200c: MARCKS [39], SEC23A [20, 24], FSCN1 [20], and CFL2 [17, 20, 24], implicating that our strategy was valid to identify *bona fide* miR-200c targets. For FSCN1, this is the first study to show regulation by miR-200c on protein level. To further validate the identified proteins, we used *in silico* target prediction to identify possible direct targets of miR-200c. Three of the four known targets were predicted by all used prediction algorithms, while FSCN1 was only predicted by three out of six algorithms (Table 2).

**Table 2:** *In silico* evaluation of miR-200c target candidates from the proteomic profiling experiment

Target candidate gene name	Target prediction	miR-200c binding sites	miR-SVR score	Published in
**CFL2**	6/6	1	−1.26	[17, 20, 24]
**FSCN1**	3/6	1	−1.17	[20]
**GPX4**	1/6	0	0	
**LIMK1**	2/6	1	−0.03	
**MARCKS**	6/6	3	−2.56	[39]
**PRKAR1A**	3/6	1	−0.49	
**SEC23A**	6/6	3	−2.61	[20, 24]
**TBCE**	1/6	0	0	

Target prediction was performed using six independent algorithms. Number of miR-200c binding sites and their corresponding miR-SVR score was accessed via microRNA.org.

In the same way, we employed miRNA target prediction for the four remaining proteins. GPX4 and TBCE were predicted by only one out of six algorithms. No miR-200c binding site was identified in their 3′UTRs. Thus, even if their abundance was consistently changed in miR-200c treated cells, they are unlikely to be directly targeted by miR-200c. LIMK1 was predicted by two out of six algorithms. Analysis of the LIMK1 3′UTR revealed one miR-200c binding site (Table 2 and Figure 1C) with a poor mirSVR score (−0.03). PRKAR1A was predicted by three out of six algorithms and its 3′UTR displays a conserved binding site (Figure 1C) with a good mirSVR score (−0.48). We conclude that PRKAR1A is a *bona fide* miR-200c target, while LIMK1 remains a target candidate. Proteotypic peptides of PRKAR1A, LIMK1 and CFL2 that were identified and quantified by mass spectrometry are depicted in Figure 1D.

Interestingly, both PKA and LIMK1 were recently described to regulate cofilin activity, thereby controlling cell migration in murine embryonic fibroblasts [29]. Regulation of cofilin phosphorylation by PKA and LIMK1 is depicted schematically in Figure 1E. The cofilin pathway plays a central role in actin filament remodeling which is essential for chemotaxis, cell migration, and invasion of cancer cells [40]. Furthermore, CFL2 regulation has been shown to be a crucial step in miR-200c induced migration inhibition [20]. Given the importance of CFL2 targeting for mediating the effects of miR-200c, it seems striking that upstream regulators of cofilins are targeted at the same time.

In previous studies, PRKAR1A has been reported to be overexpressed in a wide array of cancer types, and to be correlated with poor prognosis in cancer patients [41]. Antisense strategies against PRKAR1A have been used to suppress tumor malignancy in several cancer cell types [42, 43] and have been successfully applied in a combinational treatment in different tumor entities *in vivo* [44, 45].

### Identification of PRKAR1A and LIMK1 as direct targets of miR-200c

**Figure 2:**
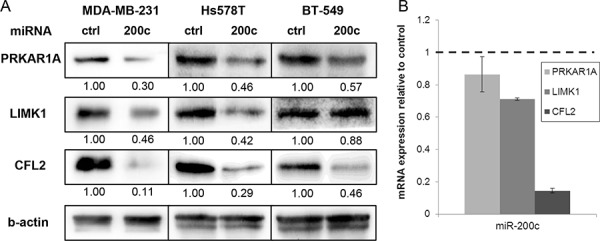
Evaluation of the miR-200c target candidates **A.** Immunoblotting of miR-200c target candidates corroborates the changes in protein abundance that were detected in the proteomic profiling in MDA-MB-231 cells. Similar changes in protein abundance were observed in the mesenchymal breast cancer cell lines Hs578T and BT-549. Relative band intensity is given below he images. **B.** mRNA level of miR-200c target candidates were evaluated by qPCR in MDA-MB-231 cells. Expression was normalized to *b-actin* and is relative to miR-ctrl treated cells.

To verify the described changes in protein abundance, we corroborated our mass spectrometry results by immunoblotting (Figure 2A). We confirmed reduction of PRKAR1A, LIMK1, and CFL2. To exclude a cell line specific effect, we analyzed two additional triple-negative breast cancer cell lines, namely BT-549 and Hs578T, by immunoblotting. Both lines display a mesenchymal phenotype and low expression of miR-200c [18]. After transfection of miR-200c, we detected reduced amounts of PRKAR1A and CFL2 in both cell lines, while LIMK1 was only reduced in Hs578T, but not in BT-549.

To complement our findings, we measured mRNA expression of CFL2, LIMK1, and PRKAR1A in MDA-MB-231 cells by qPCR (Figure 2B). CFL2 expression was strongly reduced after miR-200c transfection, corroborating the results on protein level and arguing for miR-200c mediated mRNA degradation. For LIMK1, mRNA levels were slightly reduced in MDA-MB-231, which was reflected in reduced LIMK1 protein abundance. Interestingly, for PRKAR1A, mRNA level was unaltered, although the protein amount was decreased and there is a miR-200c binding site in the mRNA 3′UTR. The unchanged mRNA level after miR-200c transfection implicates regulation by translation inhibition rather than degradation.

In summary, we identified several proteins of the PKA-LIMK1-cofilin pathway to be downregulated after miR-200c transfection of MDA-MB-231 cells in a proteomic profiling approach. Bioinformatic analysis supported that PRKAR1A and LIMK1 are targets of miR-200c. PRKAR1A was consistently reduced in protein amount in three different cell lines, as assessed by immunoblotting. Analysis of the mRNA level revealed a degradation independent mode of translation inhibition for PRKAR1A. LIMK1 protein amount was reduced in two of three cell lines, accompanied by decreased mRNA level in MDA-MB-231 and identification of a miR-200c binding site in the 3′UTR. However, LIMK1 seems to be a weaker target than PRKAR1A, as reflected in less prominent reduction of protein and mRNA amount.

### miR-200c influences both regulatory and catalytic subunits of PKA

In our proteomic profiling, we found miR-200c dependent reduction in a regulatory subunit of the PKA enzyme. PKA is a heterotetramer of two regulatory (R) and two catalytic (C) subunits. In the inactive state, the catalytic subunits are bound and inhibited by the regulatory subunits. Upon cAMP-binding to the regulatory subunits, the catalytic subunits are released from the complex and free to phosphorylate target proteins. Four different isoforms of regulatory subunits (PRKAR1A, PRKAR1B, PRKAR2A, PRKAR2B) and three isoforms of catalytic subunits (PRKACA, PRKACB, PRKACG) have been described.

**Figure 3:**
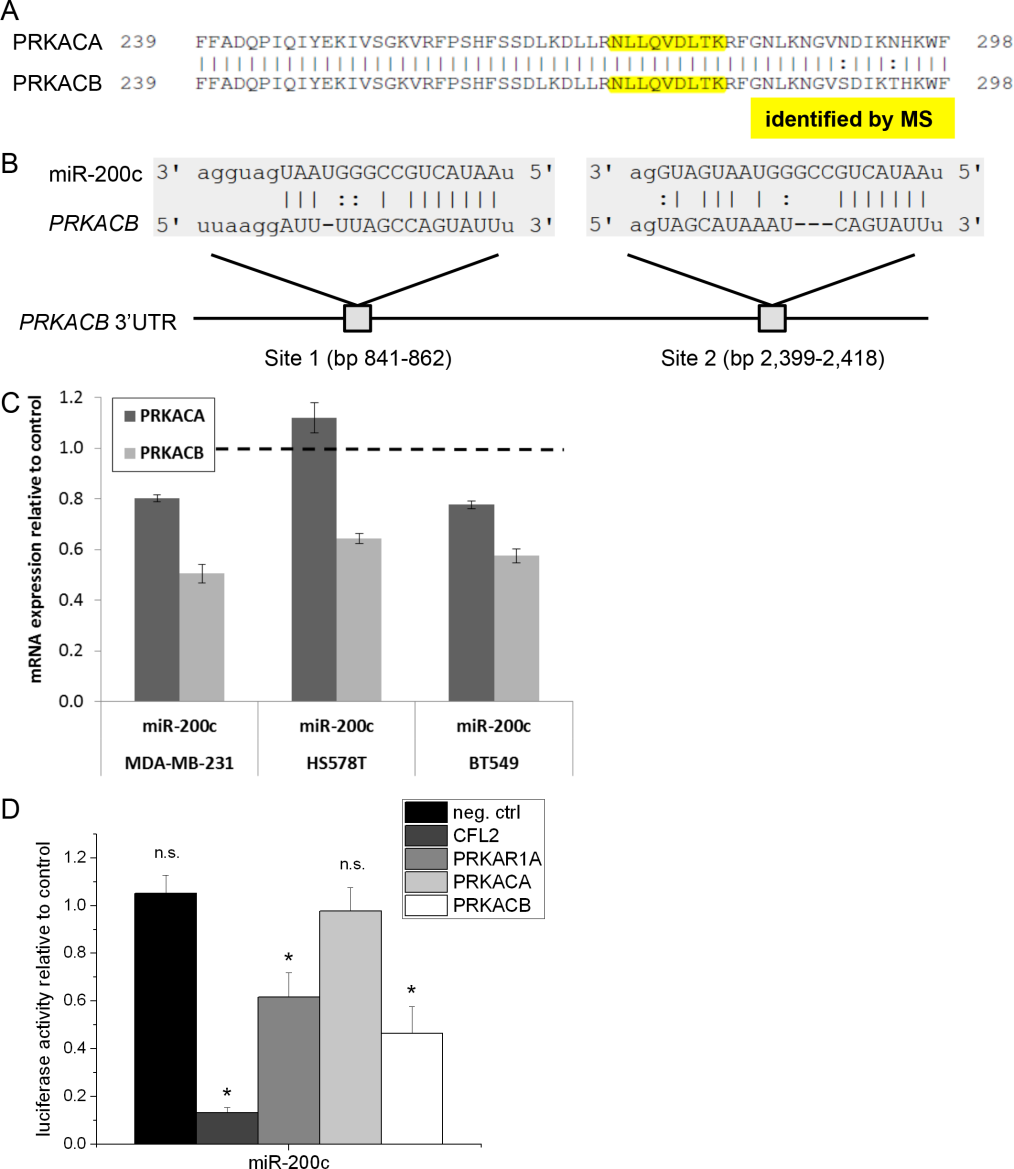
*PRKACB* is a direct target of miR-200c **A.** Partial alignment of the PKA catalytic subunits a and b proteins. Both subunits share a high sequence identity on protein level. The tryptic peptide identified by mass spectrometry (marked in yellow) does not uniquely identify one of the subunits. **B.** Two conserved miR-200c binding sites locate to the 3′UTR of *PRKACB* mRNA. In *PRKACA*, no binding site was identified. **C.** qPCR analysis of *PRKACA* and *PRKACB* mRNA level. *PRKACB* mRNA abundance is reduced to 50% of the control in MDA-MB-231, to 64% in HS578T, and to 58% in BT549. mRNA of the *PRKACA* subunit is only slightly decreased to about 80% in MDA-MB-231 and BT-549, and unaffected in Hs578T cells. (*n* = 3, one of two identical experiments shown) **D.** 3′UTR luciferase assay reveals direct targeting of *PRKAR1A* and *PRKACB*, but not *PRKACA*. *CFL2* was used as a positive control. (*n* = 8, one of two identical experiments shown).

Apart from PRKAR1A, we also identified a peptide of the catalytic PKA subunit, which was reduced in the miR-200c treated cells in one of the two biological replicates. The sequence was not unique for a single subunit, and maps to both PKA catalytic subunits α(PRKACA) and β (PRKACB) (Figure 3A). Both subunits share a sequence identity of 97.4% in the amino acid sequence, making them hard to distinguish on protein level. However, the 3′UTR of the corresponding mRNA is highly diverse, sharing only 11.3% sequence identity. To reveal possible regulation by miR-200c, we performed an *in silico* binding site analysis using the miRanda algorithm for both PKA subunits. In the 3′UTR of PRKACB, we found two conserved miR-200c binding sites at bp 841–862 and bp 2399–2418 (Figure 3B) with good mirSVR scores (−0.45 and −0.88, respectively; combined −1.33), identifying PRKACB as a potential target of miR-200c. In contrast, no binding site was identified in the 3′UTR of the PRKACA subunit. In line with that, six out of six algorithms predicted PRKACB as a miR-200c target, but only two out of six predicted PRKACA as a target. Taken together, PRKACB is more likely to be a direct target of miR-200c than PRKACA.

To corroborate these findings, we probed mRNA levels of both PRKACA and PRKACB. In line with the bioinformatic analysis, we observed that miR-200c treatment results in a strongly reduced mRNA level of PRKACB, but not of PRKACA in MDA-MB-231 cells. The same effect was seen in Hs578T and BT-549 cells (Figure 3C).

To confirm the direct binding of miR-200c, we used luciferase vectors carrying the full 3′UTRs of PRKAR1A, PRKACA, or PRKACB. We used the 3′UTR of CFL2 as a positive control. Treatment with miR-200c clearly reduced luciferase activity in the case of PRKAR1A and PRKACB, while having no effect on PRKACA (Figure 3D). These results confirmed that both subunits PRKAR1A and PRKACB are direct targets of miR-200c.

### Patient and cancer cell line expression data support a miR-200c-PKA axis

Several studies have analyzed the miRNA-200 family in the development and progression of breast cancer *in vivo* [20, 58–60]. Interestingly, depending on cellular context and tumor stage, these studies had controversial outcomes. In general, miR-200 family members are reported to have a tumor suppressive function in epithelial tumors through enforcement of epithelial characteristics and suppression of EMT [20] or repression of actin- associated genes [60].

To validate the regulation of PRKAR1A and PRKACB by miR-200c, we correlated mRNA expression of both PKA subunits to miR-200c expression in the NCI-60 panel of tumor cell lines. Both subunits displayed the expected negative correlation to miR-200c expression, with sPCC (PCC) values of −4.95 (−0.18) and −7.12 (−0.30), respectively. Thus, the regulation of the subunits by miR-200c is not restricted to breast cancer cells, but reflected in a wide array of cancer cell lines.

**Figure 4:**
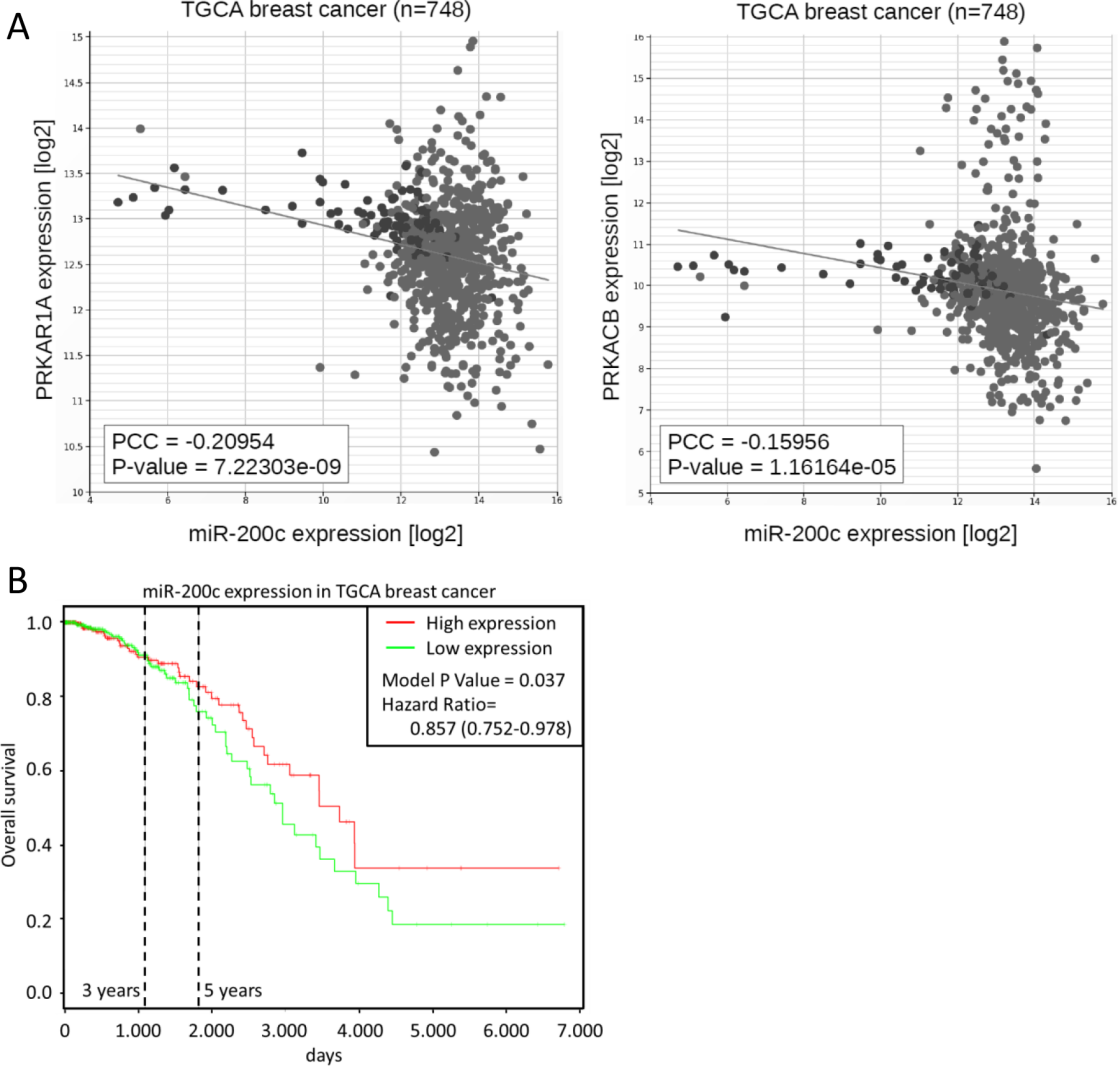
PKA subunits are correlated to miR-200c in patient data **A.** Correlation of miR-200c and *PRKAR1A* or *PRKACB* expression in 748 breast cancer patient samples of the TGCA dataset. **B.** Kaplan-Meier curve of overall survival in the TCGA breast cancer dataset. To define high and low expression, the dataset was divided at median miRNA expression.

Importantly, the negative correlation of miR-200c and both PKA subunits is also found in clinical patient data, namely data of the cancer genome atlas (TCGA). Both subunits showed a highly significant negative correlation to miR-200c in the TCGA dataset for breast cancer (Figure 4A). The TCGA data also highlights a significant (*p* < 0.05) correlation of elevated miR-200c expression and prolonged overall patient survival in breast cancer (Figure 4B). Collectively, these data strongly support a miR-200c-PKA axis in cancer biology.

### Downregulation of PKA subunits contributes to miR-200c mediated suppression of migration

Combining proteomics, bioinformatics, and cellular methods, we showed that two PKA subunits, PRKAR1A and PRKACB, are *bona fide* targets of miR-200c. To clarify the outcome of miR-200c-induced reduction of protein abundance of PKA subunits, we measured the PKA activity after miR-200c transfection using two independent methods: western-blot analysis with an antibody detecting phosphorylated R-X-X-S/T motifs and pCREB-sensitive luciferase assays reflecting phosphorylation of the CREB transcription factor, which is a known PKA target (Supplementary Figure S2A and S2B). We did not detect a significant change of overall PKA activity 48 h–72 h after miR-200c treatment, implying that the reduction in both a regulatory and a catalytic subunit of PKA resulted in a net unchanged PKA activity. However, we observed slight differences in the band pattern of PKA substrates after miR-200c treatment (Supplementary Figure S2A). PKA complex composition has a major influence on its biochemical properties and localization in the cell [46–48]. Changes in subunit abundance modify the affinity of the PKA complex towards cAMP [49] and A-kinase anchor proteins (AKAPs) [48]. The impact of specific subunits on substrate specificity has not yet been determined in detail, but it has been shown that PKA may be activated towards specific targets, without leading to an overall PKA activation [50].

To test the effect of PKA activity on the miR-200c induced cellular phenotype, we inhibited or activated PKA in parallel with miRNA transfection. PKA has long been known to influence the migratory ability of cells, although there are both reports of positive or negative influence [51]. In MDA-MB-231, inhibition of PKA activity has been shown to impede cell migration [52, 53]. In line with these earlier findings, inhibition of PKA reduced migration of miR-ctrl cells. In contrast, inhibition of PKA did not further reduce migration of cells treated with miR-200c (Supplementary Figure S2C). Contrarily, stimulation of PKA activity using the adenylat-cyclase activator forskolin (FSK) did not increase migration after miR-ctrl treatment, but fully rescued the migration inhibition by miR-200c (Supplementary Figure S2C and S2D). We conclude that miR-200c migratory inhibition can be partly reproduced by PKA inhibition and completely overcome by PKA activation.

**Figure 5:**
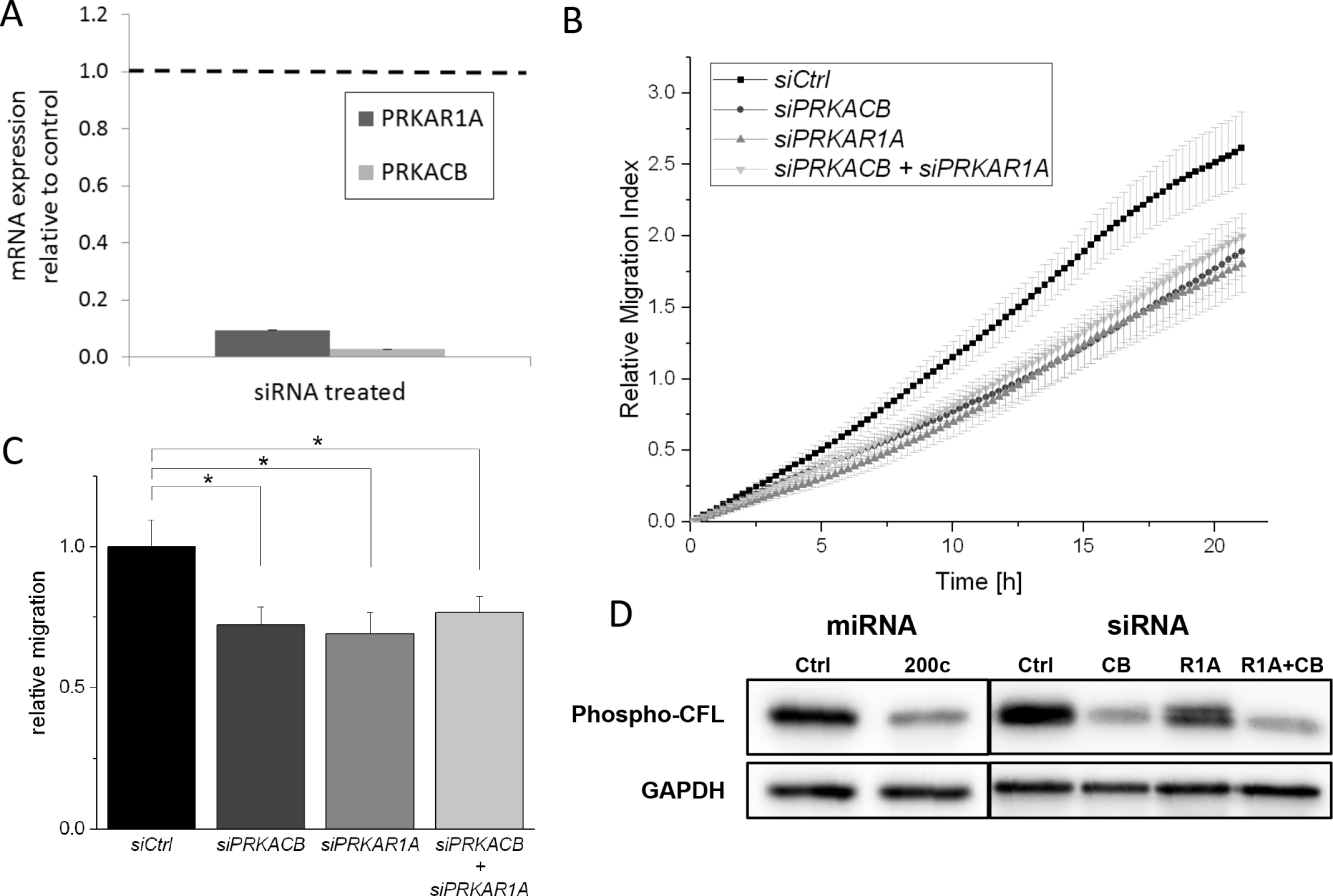
siRNA-mediated translation inhibition of PRKAR1A and PRKACB subunits reduces migration of MDA-MB-231 cells **A.** MDA-MB-231 cells were treated with siRNA targeted at *PRKAR1A* or *PRKACB*, respectively, for 48 h. Degradation of target mRNA was confirmed by qPCR. **B.** Migration of MDA-MB-231 cells was measured in a trans-well experiment after siRNA-mediated repression of PKA subunits. Migration was inhibited by both *PRKAR1A* and *PRKACB* knockdown. Combined knockdown of both subunits showed no additional effect. **C.** Endpoint values of migration assay. (Error bars depict S.E.M.; *n* > 7) **D.** Immunoblotting showed a strong reduction of phosphorylated CFL. The antibody detects both phospho-CFL1 and phospho-CFL2.

For a more detailed investigation, we employed siRNA-mediated depletion of the PKA subunits PRKAR1A and PRKACB. We optimized the siRNA transfection to achieve a decrease in expression of the intended targets (Figure 5A), while not targeting other PKA subunits (Supplementary Figure S3A and S3B). In line with the previous findings, siRNA mediated, targeted downregulation of PRKAR1A or PRKACB led to a reduction of the migratory ability of MDA-MB-231 cells (Figure 5B and 5C). A cumulative or potentiating effect upon silencing of both subunits was not observed. These results strengthen the impact of the miR-200c-PKA axis on cellular migration.

As highlighted in Figure 1E, the PKA system, through LIMK, acts on cofilin phosphorylation. Cofilin exists in two isoforms, CFL1 and CFL2, with CFL2 being a major target of miR-200c. CFL1 is expressed to a much higher degree than CFL2 [56], but is not targeted by miR-200c. In our proteomic dataset, CFL1 was identified with 4 unique peptides in both experiments, but no change of protein abundance was detected. We used the APEX method to calculate protein abundances based on our proteomic data [54, 55]. The resulting APEX scores corroborate much higher expression of CFL1 than CFL2 (Supplementary Figure S4). Using western-blot analysis with a phosphorylation-specific antibody, we assessed phosphorylation of CFL1/CFL2 in MDA-MB-231 cells upon expression of miR-200c or silencing of PRKAR1A and PRKACB. Notably, the antibody detects phosphorylation of both CFL1 and CFL2. While phosphorylation was reduced in all three conditions (Figure 5D), PRKACB silencing had a stronger impact on CFL1/CFL2 phosphorylation than PRKAR1A. A cumulative or potentiating effect upon silencing of both subunits was not observed. Due to the high abundance of CFL1, which was not affected by expression of miR-200c, we conclude that the impact of miR-200c on the PKA system leads to altered phosphorylation of CFL1. Since cofilin phosphorylation is implicated in cellular migration [40, 57], this observation links the miR-200c-PKA-LIMK axis to cellular migration through cofilin phosphorylation.

### PKA subunits and CFL2 form a miRNA target cluster

**Figure 6:**
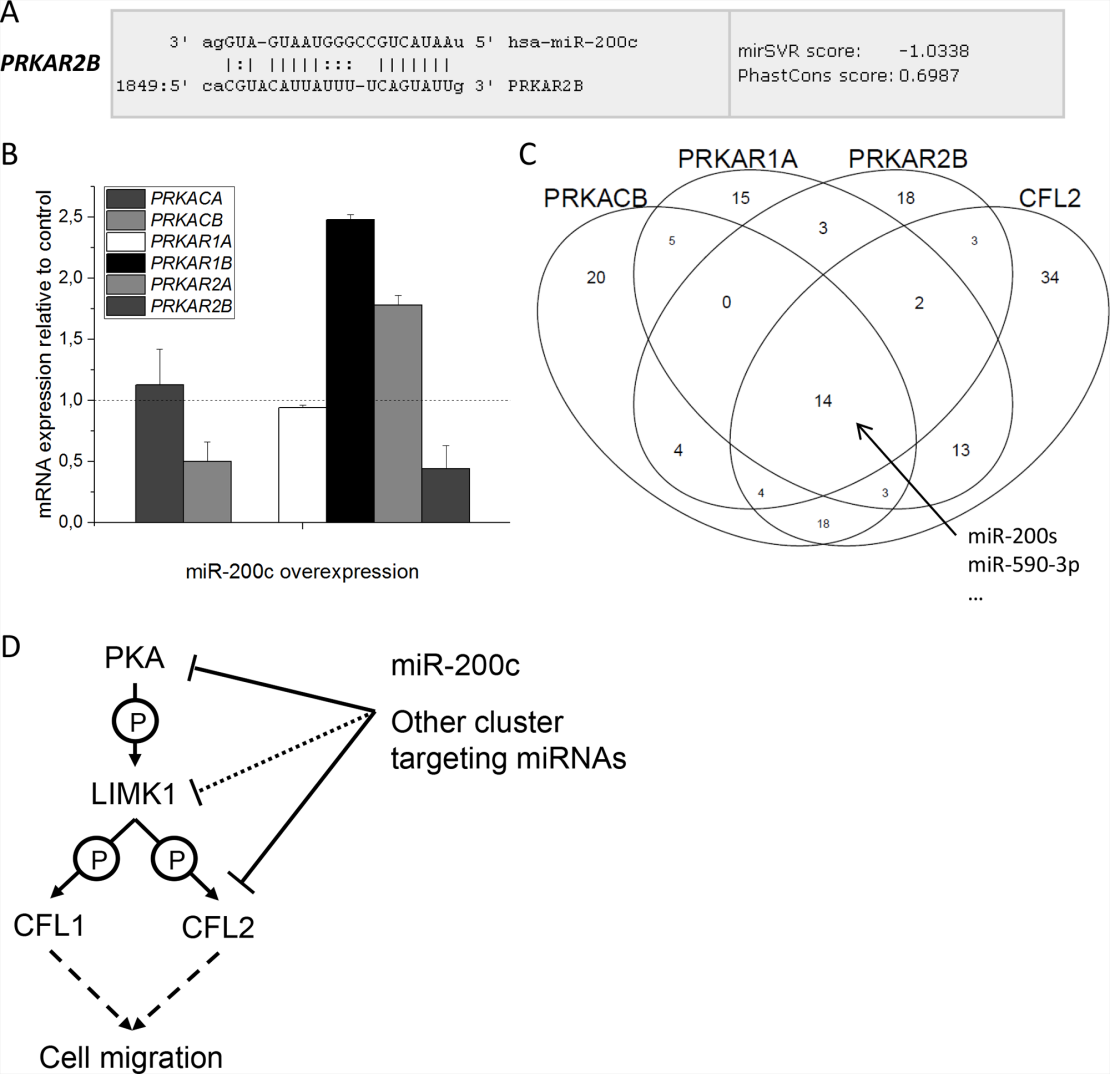
Influence of miRNA binding on PKA subunits **A.** miR-200c binding site in the 3′UTR of *PRKAR2B* mRNA. **B.** qPCR of all PKA subunits after transfection of miR-200c in MDA-MB-231 cells. *PRKACB* and *PRKAR2B* mRNA is reduced to about 50%, *PRKACA* and *PRKAR1A* are unchanged, while *PRKAR1B* and *PRKAR2A* are increased 2.5- and 2.0-fold, respectively. *PRKACG* mRNA was below detection level. (*n* = 3, one of two identical experiments shown) **C.** Overlap of different miRNA entities binding to members of the miRNA target cluster*PRKACB*, *PRKAR1A, PRKAR2B, and CFL2*. 14 different miRNAs, among them all miR-200 family members, bind to the complete cluster. Notice also the high overlap between *CFL2* and *PRKACB*, as well as *CFL2* and *PRKAR1A*. **D.** Schematic drawing of the proposed mechanism for migration inhibition, which is exerted by miR-200c and probably also other cluster targeting miRNAs.

Given the simultaneous reduction of both a regulatory, as well as a catalytic subunit of the PKA enzyme by miR-200c, we searched the microRNA.org database for all PKA subunits (Table 3). Interestingly, yet another PKA subunit, PRKAR2B (not identified in the proteomic analysis), had a strong binding site for miR-200c (Figure 6A). The mRNA level of all PKA subunits as probed by qPCR in the MDA-MB-231 cell line showed a clear downregulation of PRKAR2B mRNA after miR-200c overexpression (Figure 6B). On the contrary, the two regulatory subunits PRKAR1B and PRKAR2A that had no miR-200c binding site were upregulated on mRNA level 2.5- and 2.0-fold, respectively, most likely due to a compensatory mechanism.

**Table 3:** Bioinformatic analysis of all PKA subunits, as well as CFL1/2

Gene name	Protein identity/similarity tofirst isoform	Length of 3′UTR [bp]	# of miRNAs binding to 3′UTR
**PRKAR1A**	1.00/1.00	2, 318	55
**PRKAR1B**	0.81/0.91	680	3
**PRKAR2A**	0.35/0.50	925	28
**PRKAR2B**	0.34/0.48	2, 228	48
**PRKACA**	1.00/1.00	1, 415	10
**PRKACB**	0.93/0.95	3, 191	68
**PRKACG**	0.83/0.92	497	8
**CFL1**	1.00/1.00	520	5
**CFL2**	0.81/0.90	2, 483	91

Protein identity and similarity is relative to the first isoform, i.e. PRKAR1A for the regulatory PKA subunits, PRKACA for the catalytic PKA subunits, and CFL1 for cofilins.

When comparing the 3′UTR of all PKA subunits, we found it striking that the regulatory subunits, as well as the catalytic subunits, show a high sequence identity on protein level and in the CDS region of the mRNA, but major variations in the 3′UTR region of the mRNA (Table 3). These variations result in different miRNA binding potential. Some of the PKA subunits display a high number of miRNA binding sites, while others possess almost none (Table 3). For example, microRNA.org lists 68 different miRNAs binding to the 3′UTR of PRKACB, but only 10 miRNAs binding to PRKACA. Regulation of PKA subunit expression by miRNA binding, like we proved here for miR-200c, may therefore be an important mechanism to fine-tune PKA complex composition. A similar phenomenon was observed for the two isoforms of cofilin. Both isoforms display a high sequence identity at CDS and protein level, but diverge in the 3′UTR (Table 3). While only five miRNAs bind to the short 3′UTR of CFL1, 91 miRNAs may bind to the much longer 3′UTR of CFL2.

These findings raised the question if other miRNA entities apart from miR-200c target more than one PKA subunit at once. Analysis of the microRNA.org database revealed a set of 14 miRNAs that target PRKAR1A, PRKAR2B, PRKACB, and CFL2 simultaneously, (Figure 6C and Supplementary Table S1), among them the whole miR-200 family (miR-141, miR-200a, miR-200b, miR-200c, and miR-429). Most strikingly, all members of the target cluster displayed the strongest miR-SVR score for the same miRNA, namely miR-590-3p (Figure 6C and Supplementary Table S2). Analysis of the remaining PKA subunits revealed that this type of combined targeting is not common for other PKA subunits (Supplementary Figure S5A and S5B). In the same way, CFL1 is not targeted by this set of miRNAs (Supplementary Figure S5C).

## DISCUSSION

Through quantitative proteomic profiling of MDA-MB-231 cells after transfection of miR-200c we identified several known and potential novel miRNA targets. In addition to the previously established miR-200c target CFL2, we identified two kinases involved in the upstream signaling of cofilin, PKA and LIMK1, as novel miR-200c targets. Reduced protein abundance of the regulatory PKA subunit PRKAR1A was reproducible in other breast cancer cell lines, though mRNA levels were unaltered. This argues for a non-degradative mechanism of mRNA suppression and underlines the importance of proteomic approaches for miRNA target identification to supplement transcriptomic screens. While bioinformatic analysis identified PRKAR1A as a *bona fide* miR-200c target, LIMK1 targeting is less clear. Abundance of the LIMK1 protein and mRNA was reduced in two out of three breast cancer cell lines, but was unchanged in a third one. Thus, LIMK1 regulation by miR-200c is probably weak and might be dependent on other, cell-specific factors. The combined targeting of the kinases upstream of cofilin might be a mechanism by which miR-200c is able to control the activity of CFL1, which is no direct target (Figure 6D).

In addition to the PKA regulatory subunit PRKAR1A, we identified one of the PKA catalytic subunits to be directly targeted by miR-200c. Although the different catalytic subunits PRKACA and PRKACB cannot be distinguished on protein level, we could show by bioinformatics, confirmed by qPCR, that only PRKACB is a miR-200c target. Similar to the reduction of PRKAR1A and PRKACB, we observed an induction of PRKAR1B and PRKAR2A subunits. Thus, miR-200c overexpression changes the composition of the PKA complex. While we detected no overall reduction of PKA activity after miR-200c overexpression, silencing of the two subunits PRKACB and PRKAR1A by siRNA overexpression phenocopied the miR-200c effects on the migratory ability of MDA-MB-231 cells. Accordingly, CFL1/CFL2 phosphorylation was reduced both by miR-200c overexpression as well as by silencing of PRKACB and PRKAR1A. Thus, we identified these PKA subunits as important mediators of miR-200c induced inhibition of migration.

Recent data revealed a role of miR-200c in metastasis formation that is beyond the miR-200c/ZEB1 axis of EMT regulation. By overexpression of miR-200 family members in an orthotopic xenograft model using the MDA-MB-231 LM2 cell line, Li and colleagues proved miR-200 to regulate tumor cell plasticity and metastasis [60] via suppression of actin-related genes such as *moesin*. Importantly, miR-200c plays a seemingly paradoxical role in cancer metastasis. While stable miR-200c expression in the primary tumor prevents early steps of metastasis [60, 61], enforced expression of miR-200c may facilitate tumor cell extravasation and colonization in a context dependent manner [24, 59] for example by influencing the cancer secretome.

Analyzing general miRNA binding to all seven PKA subunits revealed that a third PKA subunit, PRKAR2B, is likely to be targeted by miR-200c directly. Furthermore, we showed that simultaneous targeting of PRKACB, PRKAR1A, PRKAR2B and CFL2 is not restricted to miR-200c, but a rather common mechanism shared by 14 different miRNAs. For this phenomenon we coined the term “miRNA target cluster”.

Strikingly, we identified miR-590-3p to have a very strong impact on the described miRNA target cluster, based on binding predictions (summed mirSVR-score of −9.77, Supplementary Table S2). Though little research has been done on miR-590-3p so far, it has been identified together with 9 other miRNAs, among those miR-200b and miR-200c, to be commonly deregulated in a wide range of cancer entities [62]. Fitting to our data, it was shown that miR-590-3p overexpression inhibits the migration of a bladder cancer cell line [63].

In conclusion, we propose the PKA enzyme as a novel target of miR-200c that mediates effects on cellular migration inhibition. *In silico* analysis revealed that PKA subunits, though very similar on protein sequence, diverge in their capability of being targeted by miRNAs. Thus, regulation of subunit expression by miRNA binding poses a new mechanism to fine-tune PKA complex composition.

## MATERIALS AND METHODS

### Cell culture and stable isotope labeling in cell culture

Human breast cancer cells lines MDA-MB-231, BT-549, and Hs578T were obtained from American Type Culture Collection (ATCC). HEK293T cells were obtained from Cell Line Services (CLS). Cells were cultured in Dulbecco's modified eagle's medium (DMEM, PAN, Aidenbach, Germany) supplemented with 10% fetal calf serum (PAN) and 1% penicillin/streptomycin stock solution (Gibco/Invitrogen, Paisley, UK) at 37°C in humidified air containing 5% CO_2_. For MS experiments, cells were cultured for two weeks in SILAC DMEM (without arginine, lysine and glutamine, high glucose [4.5 g/l]) supplemented with 10% fetal bovine serum and labeled or unlabeled arginine and lysine, respectively (Silantes, Munich, Germany).

### Transfections

Transfection of Ambion^®^ Pre-miR™ miRNA Precursors (Life Technologies GmbH) and of siRNA (Sigma-Aldrich) was performed with Lipofectamine RNAiMAX transfection reagent (Qiagen) according to the manufacturer's protocol. 180 pmol or 4.5 pmol of miRNA was used for transfection of 7.5 × 10^5^ cells (10 cm dish) or 2.5 × 10^4^ cells (24 well plate), respectively. 30 pmol of siRNA was used for transfection of 1.25 × 10^5^ cells (6 well plate). The following siRNA constructs were used: PRKACB; CGAGUACCUCCAUUCACUA (SASI_Hs01_00188721) and PRKAR1A; GAUGUAUGAGGAAUUCCUU (SASI_Hs01_00116785).

### MS sample preparation

48 hours after miRNA transfection, cells were detached by using 10 mM ethylenediaminetetraacetic acid (EDTA) and lysed in RIPA buffer (1% NP-40, 150 mM NaCl, 50 mM Tris pH7.5, 0.25% sodium deoxycholate, 5 mM EDTA, 0.01 mM E64, 1 mM PMSF). Samples were mixed 1:1 according to protein content, as determined by BCA assay (Pierce^®^ Protein Quantification Kit, Thermo Scientific) and concentrated by centrifugation in a 3 kDa Vivaspin 500 spin filter (Sartorius Stedim Biotech GmbH, Goettingen, Germany) at 15, 000 g. 4x sample buffer (NuPAGE, Invitrogen) was added and proteins were reduced using 10 mM DTT (AppliChem GmbH, Darmstadt, Germany) for 30 min at 75°C and alkylated using 20 mM IAM (Sigma-Aldrich) for 30 min at room temperature. Protein mixtures were separated by SDS–PAGE using 4–12% Bis-Tris mini gradient gels (NuPAGE, Invitrogen). The gel lanes were cut into 10 equal slices, which were in-gel digested with trypsin (Worthington, Lakewood, NJ, USA) [64], and the resulting peptide mixtures were processed on self-packed C18 STAGE tips (Empore, St. Paul, MN, USA) [65].

### LC-MS/MS and data computing

LC-MS/MS was performed as described previously [66] using an Orbitrap XL mass spectrometer (ThermoScientific GmbH, Bremen, Germany). Data computing was essentially done as described previously [35], with the following changes: For database search, the Uniprot reference human proteome dataset, retrieved at February 28, 2012, was used. The mass tolerance was 10 ppm for parent ions and 0.3 Da for fragment ions. Static modification was cysteine carboxyamidom ethylation (57.02 Da); potential modifications were heavy lysine and arginine (each 6.020129 Da).

The relative quantification for each protein was calculated from the relative areas of the extracted ion chromatograms of the precursor ions and their isotopically distinct equivalents using the XPRESS [67] and ASAPRatio [36] algorithms. Only proteins that yielded convergent quantitations in XPRESS and ASAPRatio (less than twofold divergence of both quantification algorithms, or both > 3.0 or < 0.33) were considered for further analysis.

All relevant data were uploaded to the Peptide Atlas database and can be downloaded from the hash code: http://www.peptideatlas.org/PASS/PASS00660.

### Migration assay

Migration assay was carried out using the xCELLigence System (Roche, Mannheim, Germany). The lower chamber of a CIM plate 16 (Roche) transwell plate was loaded with 160 μl of DMEM, containing 10% FCS. The upper chamber was loaded with 50 μl of DMEM without FCS. The transwell plate was equilibrated at 37°C for one hour. Cells were detached and 2 × 10^4^ cells in 100 μl DMEM per well were seeded into the upper chamber. Cell migration was monitored in real time by measuring the electrical impedance across interdigital gold microelectrodes in the lower chamber. Migration was measured 48 h to 72 h after miRNA transfection. The migration index was normalized to a timepoint 3 h after beginning the migration experiment. Migration was measured every 15 min for 24 h. Each experiment was performed in quadruplicates and outliers were removed, if necessary. Curves of migration experiments depict one typical experiment, while endpoint assays contain the data of all experiments.

### Immunoblotting

Immunoblotting was performed as described previously [66]. The following antibodies were used: LIMK1 (BD Transduction Laboratories, #611748), PRKAR1A (BD, #610609), PRKACA/CB (BD, #610980), CFL2 (Abcam, ab39985), CFL phospho S3 (Cell Signaling, #3311). Blots were normalized to β-actin (MP Biomedicals, #69100). Quantification of bands was performed using the ImageJ software (v4.18).

### Bioinformatics

miR-200c target prediction was performed using miRecords [68], which is available at http://mirecords.umn.edu/miRecords. Binding site prediction was performed using the miRanda algorithm [69] accessed at microRNA.org [70]. Correlation of miRNA and mRNA expression in the NCI60-panel was performed using miRConnectL [71], available at mirconnect.org. Alignments were performed using Needle, accessed at http://www.ebi.ac.uk/Tools/. mRNA sequence analysis was performed using the predominant transcript (variant 1) of each gene, accessed at NCBI Nucleotide on 150119, protein sequence analysis was performed using the isoform 1, accessed at Uniprot on 150119. Kaplan-Meier curves and mRNA-miRNA correlation using the TCGA BRCA dataset were calculated and plotted using PROGmiR [72] and starBase v2.0 [73], respectively.

### 3′UTR luciferase assay

For 3′UTR luciferase assay, we used pEZX-MT05 vectors (Genecopoeia, Rockville, USA) containing the mRNA 3′UTRs of the possible target proteins. Following constructs were used: CmiT000001-MT05 (control vector), HmiT014575 (PRKACA), HmiT055226 (CFL2), HmiT014584 (PRKACB), HmiT059816 (PRKAR1A). HEK293T cells were co-transfected with 50 ng plasmid and 50 μM miRNA in a 96 well plate using Attractene transfection reagent (Qiagen) according to the provided protocol. Luciferase and alkaline phosphatase activity was measured in cell supernatants after 48 h using Gaussia Juice and SEAP Juice (p.j.k, Kleinblittersdorf, Germany), respectively. Luminescence was measured in duplicates using a Centro LB 960 luminometer (Berthold Technologies). Gaussia luciferase activity was normalized to SEAP activity.

### qPCR

Isolation of mRNA, reverse transcription, and qPCR were carried out as described previously [74]. The following primer pairs were used:

PRKACA fwd: cgggaaccactatgccatga, rev: gcgcttttcattcag ggtgt

PRKACB fwd: caagtggtttgccacgacag, rev: tgctggtatctccag agcct

CFL2 fwd: tattctgggctcctgaaagtgc, rev: ccaagtgtcgaacggt cctt

LIMK1 fwd: atggcctacctccactccat, rev: ccacattcttgttctcg cgg

PRKAR1A fwd: agcaggagagcgtgaaagaat, rev: tccaagtgggct gtgttctg

### Statistics

All samples were tested for normal distribution using the Shapiro-Wilk test. When the null hypothesis could not be rejected, samples were tested for unequal mean using two-sided Student's *t*-test. Asterisks in figures mark significantly unequal mean values of the indicated samples (*p* < 0.01), if not stated otherwise. Error bars in figures represent standard deviation, if not stated otherwise.

## SUPPLEMENTARY METHODS


