# Supplementary material for: miR-200c dampens cancer cell migration via regulation of protein kinase a subunits
Source: Oncotarget. 2015 Jun 29;6(27):23874–89. doi: 10.18632/oncotarget.4381 (PMC4695158; doi:10.18632/oncotarget.4381)
Supplement: Supplementary file 1 [file oncotarget-06-23874-s001.pdf]

SUPPLEMENTARY METHODS

PKA activity measurement

PKA activity was measured using the PathDetect CRE cis-Reporting System (Agilent). Cells were co-transfected with 400 ng pCre-Luc reporter plasmid, 8 ng pCMV-Renilla control plasmid, and 4.5 pmol miRNA using Attractene transfection reagent (Qiagen). As a positive control, cells were co-transfected with 25 ng pCMV-PRKACA. Cells were lysed on-plate (250 mM KCl, 50 mM Tris, 10% Glycerin, 0.1% NP40, pH 7.8) and luciferase activity was measured in firefly buffer (25 mM Tris, 10 mM $MgSO_4$, 2 mM ATP, 0.05 mM D-luciferin, pH 7.8) or renilla buffer (25 mM Tris, 100 mM NaCl, 1 mM EDTA, coelenterazine, pH 7.6). Luminescence was measured in duplicates using a Centro LB 960 luminometer (Berthold Technologies). Firefly luciferase activity was normalized to renilla luciferase activity.

Determination of protein abundance by the APEX method

Protein abundances in proteomic datasets were calculated with the APEX Quantitative Proteomics Tool v1.1.0 with standard settings. Training data were built from a dataset of unmodified MDA-MB-231 cell lysate, using the top 50 proteins with the highest number of identified peptides.

SUPPLEMENTARY FIGURES AND TABLES

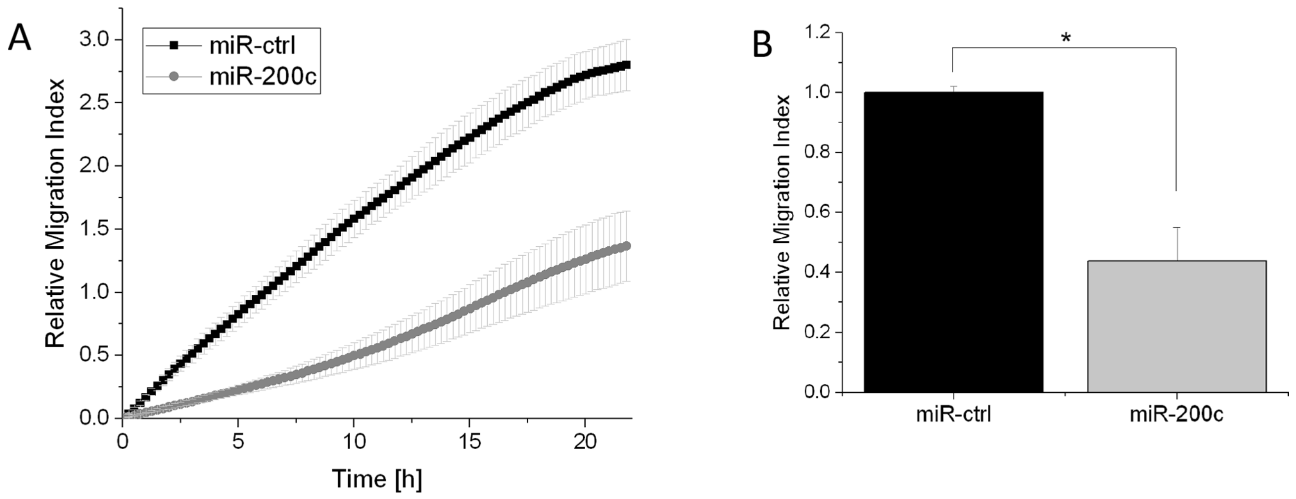

Supplementary Figure S1: Treatment with miR-200c inhibits migration ability of MDA-MB-231. A. MDA-MB-231 transfected with miR-200c displayed a strongly reduced migration capacity in a transwell assay (n = 5, one of four similar experiments shown). **B.** Endpoint values of migration of the same assay (n = 14).

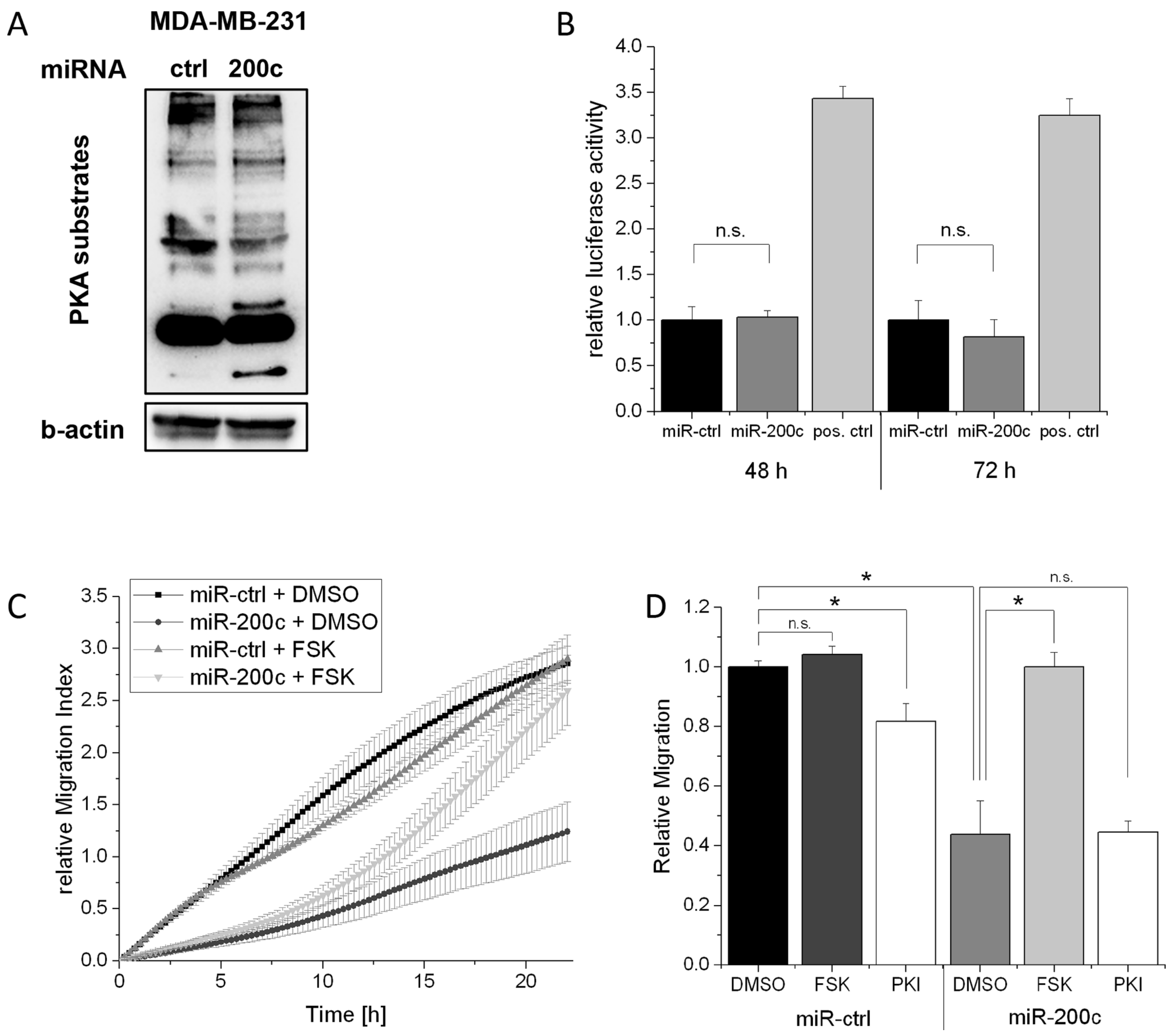

Supplementary Figure S2: PKA activity is not affected by miR-200c transfection. **A.** MDA-MB-231 cell lysates were probed with an antibody recognizing the phosphorylation motif of PKA and PKC (R-X-X-pS/pT). Although there is some variation in the phosphorylation patterns, overall kinase activity is unchanged after miR-200c transfection. **B.** MDA-MB-231 were co-transfected with a pCREB-sensitive luciferase plasmid and miR-200c. No change of luciferase activity could be detected. **C.** MDA-MB-231 were transfected with miRNA and treated either with solvent control (DMSO) or FSK (20 µM). Treatment with FSK completely restored the cellular migration capacity in cells treated with miR-200c, but had no effect on cells transfected with miR-ctrl. **D.** Endpoint values of migration assay. Cells were treated either with solvent control (DMSO), FSK (20 µM), or PKI (5 µM). PKA inhibition led to a reduction of migration in cells transfected with miR-ctrl, but did not further reduce migration of cells transfected with miR-200c.

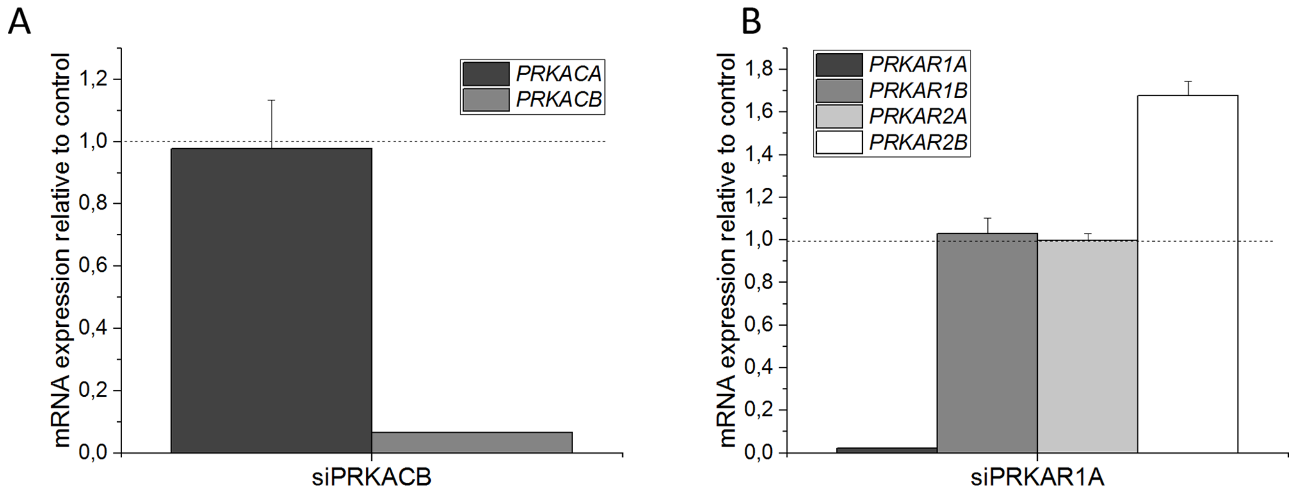

Supplementary Figure S3: siRNA-mediated knockdown of *PRKACB* and *PRKAR1A* has little effect on other PKA subunits. A. MDA-MB-231 cells were transfected with siRNA targeted at *PRKACB*. Analysis on mRNA abundance by qPCR showed that none of the other catalytic subunits were affected. **B.** MDA-MB-231 cells were transfected with siRNA targeted at *PRKAR1A*. Analysis on mRNA abundance by qPCR showed that the subunits *PRKAR1B* and *PRKAR2A* were not affected by the treatment, while expression of *PRKAR2B* is increased.

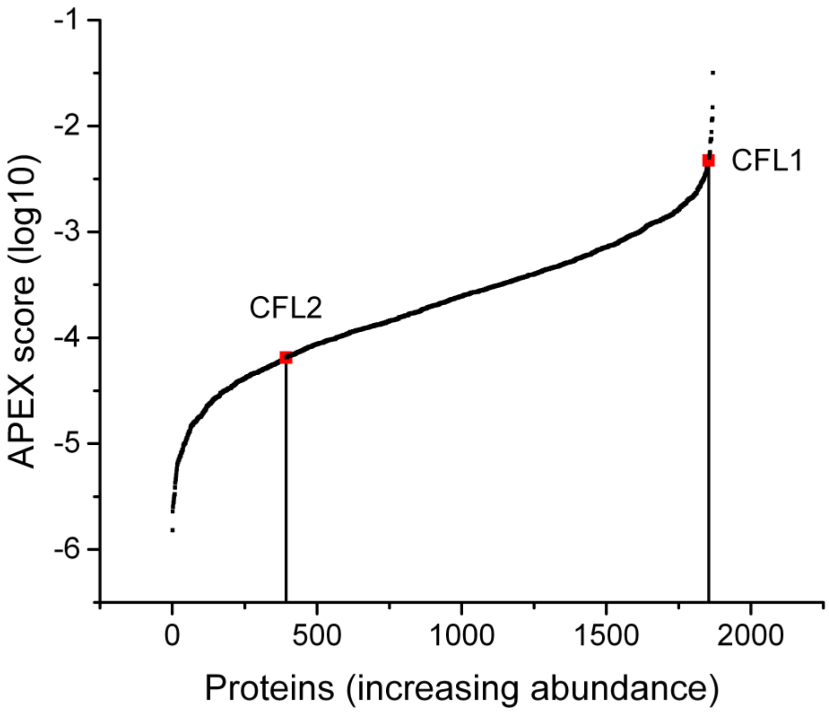

Supplementary Figure S4: Abundance of CFL1/2 proteins in MDA-MDA-231 cells was determined using APEX software. CFL1 was identified with an APEX score (log10) of −2.3, ranging in the top 1% of expressed proteins. CFL2 was identified with an APEX score (log10) of −4.2, ranging in the lowest 25% of expressed proteins.

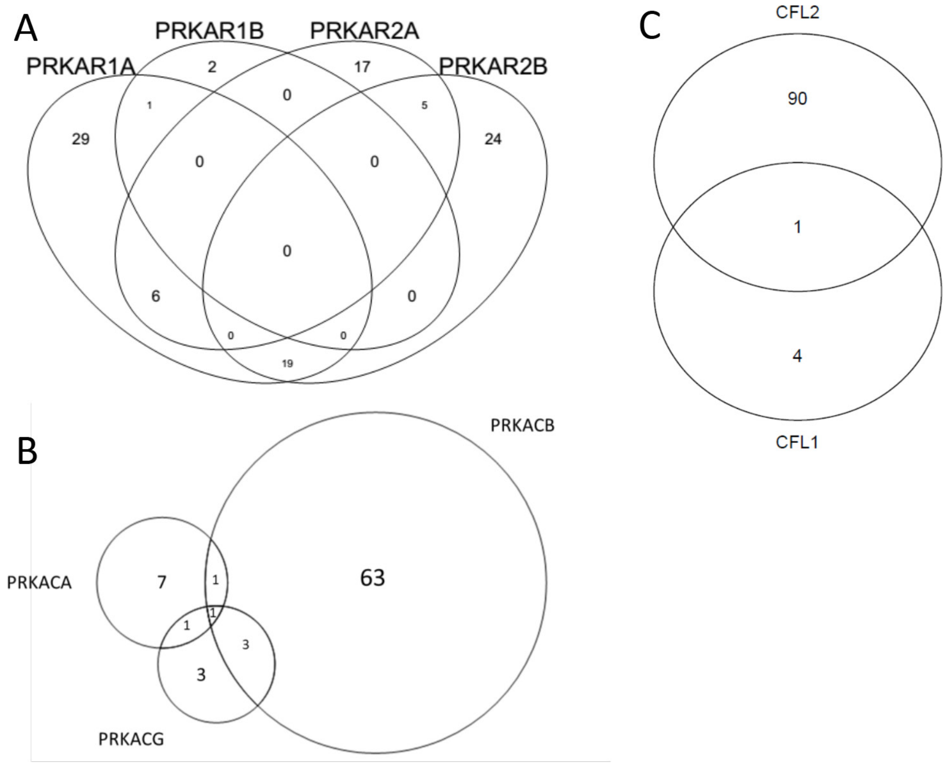

Supplementary Figure S5: Collective miRNA targeting of PKA subunits is not a general phenomenon, but specific for the group PRKACB, PRKAR1A and PRKAR2B. A. Overlap of miRNA targeting regulatory PKA subunits. **B.** Overlap of miRNA targeting catalytic PKA subunits. **C.** Overlap of miRNA targeting cofilin isoforms.

Supplementary Table S1: Complete record of miRNA entities binding to at least one member of the miRNA target cluster. Values are binary (0 = no binding site, 1 = at least one binding site).

miRNA	count	PRKACB	PRKAR1A	PRKAR2B	CFL2
hsa-miR-495	4	1	1	1	1
hsa-miR-519d	4	1	1	1	1
hsa-miR-20b	4	1	1	1	1
hsa-miR-20a	4	1	1	1	1
hsa-miR-17	4	1	1	1	1
hsa-miR-106b	4	1	1	1	1
hsa-miR-106a	4	1	1	1	1
hsa-miR-93	4	1	1	1	1
hsa-miR-141	4	1	1	1	1
hsa-miR-200a	4	1	1	1	1
hsa-miR-200c	4	1	1	1	1
hsa-miR-200b	4	1	1	1	1
hsa-miR-429	4	1	1	1	1
hsa-miR-590-3p	4	1	1	1	1
hsa-miR-410	3	0	1	1	1
hsa-miR-499-5p	3	0	1	1	1
hsa-miR-211	3	1	1	0	1
hsa-miR-204	3	1	1	0	1
hsa-miR-183	3	1	1	0	1
hsa-miR-361-5p	3	1	0	1	1
hsa-miR-186	3	1	0	1	1
hsa-miR-381	3	1	0	1	1
hsa-miR-300	3	1	0	1	1
hsa-miR-340	2	0	0	1	1
hsa-miR-448	2	0	0	1	1
hsa-miR-217	2	0	0	1	1
hsa-miR-342-3p	2	0	1	0	1
hsa-miR-320b	2	0	1	0	1
hsa-miR-320a	2	0	1	0	1
hsa-miR-320d	2	0	1	0	1
hsa-miR-320c	2	0	1	0	1
hsa-miR-107	2	0	1	1	0
hsa-miR-103	2	0	1	1	0
hsa-miR-30e	2	0	1	0	1
hsa-miR-30d	2	0	1	0	1

(*Continued*)

miRNA	count	PRKACB	PRKAR1A	PRKAR2B	CFL2
hsa-miR-30c	2	0	1	0	1
hsa-miR-30b	2	0	1	0	1
hsa-miR-30a	2	0	1	0	1
hsa-miR-194	2	0	1	0	1
hsa-miR-216a	2	0	1	0	1
hsa-miR-494	2	0	1	0	1
hsa-miR-96	2	0	1	1	0
hsa-miR-543	2	1	0	1	0
hsa-miR-421	2	1	0	0	1
hsa-miR-215	2	1	1	0	0
hsa-miR-192	2	1	1	0	0
hsa-miR-145	2	1	0	0	1
hsa-miR-383	2	1	1	0	0
hsa-miR-223	2	1	1	0	0
hsa-miR-153	2	1	0	0	1
hsa-miR-384	2	1	0	1	0
hsa-miR-372	2	1	0	0	1
hsa-miR-373	2	1	0	0	1
hsa-miR-520e	2	1	0	0	1
hsa-miR-520d-3p	2	1	0	0	1
hsa-miR-520c-3p	2	1	0	0	1
hsa-miR-520b	2	1	0	0	1
hsa-miR-520a-3p	2	1	0	0	1
hsa-miR-302d	2	1	0	0	1
hsa-miR-302c	2	1	0	0	1
hsa-miR-302b	2	1	0	0	1
hsa-miR-302a	2	1	0	0	1
hsa-miR-302e	2	1	0	0	1
hsa-miR-301b	2	1	0	0	1
hsa-miR-19b	2	1	0	0	1
hsa-miR-19a	2	1	0	0	1
hsa-miR-329	2	1	0	1	0
hsa-miR-362-3p	2	1	0	1	0
hsa-miR-182	2	1	1	0	0
hsa-miR-29c	1	0	0	0	1
hsa-miR-29b	1	0	0	0	1
hsa-miR-29a	1	0	0	0	1

(*Continued*)

miRNA	count	PRKACB	PRKAR1A	PRKAR2B	CFL2
hsa-miR-203	1	0	0	0	1
hsa-miR-506	1	0	0	0	1
hsa-miR-124	1	0	0	0	1
hsa-miR-219-5p	1	0	0	0	1
hsa-miR-491-5p	1	0	0	0	1
hsa-miR-377	1	0	0	0	1
hsa-miR-590-5p	1	0	0	0	1
hsa-miR-21	1	0	0	0	1
hsa-miR-382	1	0	0	0	1
hsa-miR-181d	1	0	0	0	1
hsa-miR-181b	1	0	0	0	1
hsa-miR-181c	1	0	0	0	1
hsa-miR-27b	1	0	0	0	1
hsa-miR-27a	1	0	0	0	1
hsa-miR-142-3p	1	0	0	0	1
hsa-miR-23b	1	0	0	0	1
hsa-miR-23a	1	0	0	0	1
hsa-miR-205	1	0	0	0	1
hsa-miR-216b	1	0	0	0	1
hsa-miR-144	1	0	0	0	1
hsa-miR-132	1	0	0	0	1
hsa-miR-212	1	0	0	0	1
hsa-miR-425	1	0	0	0	1
hsa-miR-301a	1	0	0	0	1
hsa-miR-152	1	0	0	0	1
hsa-miR-148b	1	0	0	0	1
hsa-miR-148a	1	0	0	0	1
hsa-miR-181a	1	0	0	0	1
hsa-miR-130a	1	0	0	0	1
hsa-miR-454	1	0	0	0	1
hsa-miR-130b	1	0	0	0	1
hsa-miR-296-3p	1	0	0	1	0
hsa-miR-191	1	0	0	1	0
hsa-miR-32	1	0	0	1	0
hsa-miR-367	1	0	0	1	0
hsa-miR-363	1	0	0	1	0
hsa-miR-25	1	0	0	1	0

(*Continued*)

miRNA	count	PRKACB	PRKAR1A	PRKAR2B	CFL2
hsa-miR-92b	1	0	0	1	0
hsa-miR-92a	1	0	0	1	0
hsa-miR-544	1	0	0	1	0
hsa-miR-431	1	0	0	1	0
hsa-miR-185	1	0	0	1	0
hsa-miR-9	1	0	0	1	0
hsa-miR-376c	1	0	0	1	0
hsa-miR-137	1	0	0	1	0
hsa-miR-758	1	0	0	1	0
hsa-miR-135b	1	0	0	1	0
hsa-miR-135a	1	0	0	1	0
hsa-miR-218	1	0	0	1	0
hsa-miR-196b	1	0	1	0	0
hsa-miR-196a	1	0	1	0	0
hsa-miR-208a	1	0	1	0	0
hsa-miR-208b	1	0	1	0	0
hsa-miR-424	1	0	1	0	0
hsa-miR-195	1	0	1	0	0
hsa-miR-16	1	0	1	0	0
hsa-miR-497	1	0	1	0	0
hsa-miR-15b	1	0	1	0	0
hsa-miR-15a	1	0	1	0	0
hsa-miR-155	1	0	1	0	0
hsa-miR-214	1	0	1	0	0
hsa-miR-539	1	0	1	0	0
hsa-miR-150	1	0	1	0	0
hsa-miR-1271	1	0	1	0	0
hsa-miR-1297	1	1	0	0	0
hsa-miR-26b	1	1	0	0	0
hsa-miR-26a	1	1	0	0	0
hsa-miR-193b	1	1	0	0	0
hsa-miR-193a-3p	1	1	0	0	0
hsa-miR-146b-5p	1	1	0	0	0
hsa-miR-146a	1	1	0	0	0
hsa-miR-154	1	1	0	0	0
hsa-miR-139-5p	1	1	0	0	0
hsa-miR-485-5p	1	1	0	0	0

(*Continued*)

miRNA	count	PRKACB	PRKAR1A	PRKAR2B	CFL2
hsa-miR-613	1	1	0	0	0
hsa-miR-1	1	1	0	0	0
hsa-miR-206	1	1	0	0	0
hsa-miR-370	1	1	0	0	0
hsa-miR-18b	1	1	0	0	0
hsa-miR-18a	1	1	0	0	0
hsa-miR-875-5p	1	1	0	0	0
hsa-miR-496	1	1	0	0	0
hsa-miR-376b	1	1	0	0	0
hsa-miR-376a	1	1	0	0	0

Supplementary Table S2: List of the 14 miRNA entities that bind to all members of the miRNA target cluster. Values give the summed mirSVR-score for all binding site of one particular miRNA. The list is sorted for strongest binding to the target cluster.

miRNA	Sum	PRKAR1A	PRKAR2B	PRKACB	CFL2
hsa-miR-590-3p	−9.7722	−1.5708	−2.7823	−3.3288	−2.0903
hsa-miR-200b	−4.1421	−0.488	−1.0338	−1.3591	−1.2612
hsa-miR-429	−4.1404	−0.4846	−1.0366	−1.3591	−1.2601
hsa-miR-200c	−4.116	−0.488	−1.0338	−1.333	−1.2612
hsa-miR-200a	−3.2938	−0.398	−0.9803	−1.2273	−0.6882
hsa-miR-141	−3.2823	−0.3949	−0.9803	−1.2189	−0.6882
hsa-miR-93	−3.0391	−0.6348	−0.1168	−0.6149	−1.6726
hsa-miR-20b	−3.0136	−0.6311	−0.1145	−0.6075	−1.6605
hsa-miR-20a	−3.0136	−0.6311	−0.1145	−0.6075	−1.6605
hsa-miR-17	−3.0134	−0.6274	−0.1156	−0.6075	−1.6629
hsa-miR-106b	−3.0134	−0.6274	−0.1156	−0.6075	−1.6629
hsa-miR-106a	−3.0134	−0.6274	−0.1156	−0.6075	−1.6629
hsa-miR-519d	−3.0111	−0.6237	−0.1122	−0.6038	−1.6714
hsa-miR-495	−2.4069	−0.1822	−0.912	−0.3929	−0.9198
